# Apoptosis induced by Pseudomonas aeruginosa: a lonely killer?

**DOI:** 10.1111/1751-7915.12144

**Published:** 2014-07-14

**Authors:** Alexis Broquet, Karim Asehnoune

**Affiliations:** 1 Faculté de Medicine, Laboratoire UPRES EA 3826, Université de NantesNantes, France; 2 Pôle Anesthésie Réanimations, Service d'Anesthésie Réanimation Chirurgicale, Hôtel Dieu, CHU NantesNantes, France

Apoptosis is a fundamental biological process allowing tissue homeostasis through the regulation of cell populations by eliminating unnecessary elements. During infection, pathogens have evolved to take advantage of this process for their own and are able to induce the apoptosis of cells, i.e. immune cells by the host itself.

*Pseudomonas aeruginosa* is one of the most studied opportunistic bacteria due to its significant involvement worldwide in pneumonia, corneal infections and wound burns. Several research groups have pointed out the ability of these bacteria to interfere and/or evade host immune system by inducing apoptosis of the targeted cells. In May 2014, looking up ‘*Pseudomonas aeruginosa*’ and ‘apoptosis’ keywords in PubMed search engine retrieve more than 300 hits. *Pseudomonas aeruginosa* seems to induce apoptosis through direct interaction with the host cells (the most studied system being the type-III secretion system: T3SS) or through secreting factors such as pyocyanin.

T3SS, the most well-studied virulence apparatus of *P. aeruginosa* is composed of a needle complex through which exoenzymes are injected into the host cells (Galle *et al*., 2012,2012). Recently, Beyaert's laboratory described an exotoxin-independent function of the T3SS in the killing of macrophages in an acute lung infection model (Galle *et al*., 2012,2012). Although, T3SS is a major virulence system, it is not fully required for the bacteria to display virulence as T3SS negative strains are shown to exihibit signficant virulence (example of Elsen's paper). In short, a wide variety of *P. aeruginosa* virulence factors are involved in inducing apoptosis by several distinct mechanisms, from the activation of the mitochondrial pathway, the generation of reactive oxygen species to the activation of the caspase pathways (Table 1).

**Table 1:** Virulence factors inducing apoptosis in targeted host cells (non-exhaustive list)

Virulence factors	Model used	Apoptosis pathway	Reference
Type-III secretion system (T3SS)			
Injector/needle complex	Macrophages/neutrophils	Caspase 3	Galle *et al*., 2012,2012
Exoenzymes (ExoS, T, U, Y)	Macrophages	Caspase 3	Galle *et al*., 2008
	Epithelial cells/fibroblasts	Mitochondrial pathway, caspase 3	Shafikhani *et al*., 2008
Secreted virulence factors			
Pyocyanin	Neutrophils	Reactive oxygen intermediate, cAMP	Usher *et al*., 2002
Exotoxin A	MEFs	Bak pathway	Du *et al*., 2010
Protease	Macrophages	Caspase 3	Zhang *et al*., 2003
ExlA	HUVECs endothelial cells	Unknown	Elsen *et al*., 2014

cAMP, cyclic adenosine monophosphate; HUVECs, human umbilical veins; MEFs, mouse embryonic fibroblasts.

The ability of *P. aeruginosa* to induce apoptosis in various *in vitro* model of infection (macrophages, neutrophils, epithelial cells …) or *in vivo* models such as lungs, cornea and burn wounds infections is not mediated by a single bacterial cell but rather by a multicellular population of *P. aeruginosa*. Members of such population interact with each other through a number of chemical signals known globally on for quorum sensing (QS). Quoting Rutherford and Bassler (2012), ‘Quorum sensing is a bacterial cell-cell communication process that involves the production, detection, and response to extracellular signaling molecules called autoinducers’. QS molecules were shown to regulate virulence factors such as toxins, exotoxin A, pyocyanin, … and so *in fine* apoptosis (Rutherford and Bassler, 2012). The best described QS signalling systems in *P. aeruginosa* are the N-acyl homoserine lactones systems Las and Rhl. The Las system produces and responds to N-oxododecanoyl homoserine lactone and the Rhl system to N-butanoyl homoserine lactone respectively. Las system is known to control the production of various virulence factors involved in host cell damages such as exotoxin A (Jones *et al*., 1993). On the other hand, Rhl system was described to repress the expression of genes responsible for the assembly and function of the T3SS (Bleves *et al*., 2005).

Last but not least, QS molecule such as 3-oxododecanoyl-L-homoserine lactone (3-oxo-C12-HSL) itself has been shown to induce apoptosis. Several studies have demonstrated that incubation of different cell lines with 3-oxo-C12-HSL molecule resulted in the induction of apoptosis involving calcium signalling, the mitochondrial pathway and caspase activations (Table 2). Interestingly, N-butanoyl-L-homoserine lactone (known as C4-HSL, the second major QS molecule in *P. aeruginosa*) harbouring a shorter fatty acid chain has not been shown to induce apoptosis compared with 3-oxo-C12-HSL (Tateda *et al*., 2003; Holban *et al*., 2014).

**Table 2:** Apoptosis pathways activated by 3-oxo-C12-HSL (non-exhaustive list)

Quorum-sensing molecule	Abbreviation used in the study	Model used	Apoptosis pathway involved	Reference
N-3-(oxododecanoyl)-l-homoserine lactone	3-oxo-C12-HSL	Macrophages/neutrophils	Caspase 3/8	Tateda *et al*., 2003
	OdDHL	Breast cancer cell lines	JAK/STAT pathway	Li *et al*., 2004
	OdDHL	Jurkat cell line	Mitochondrial pathway	Jacobi *et al*., 2009
	C12	Airway epithelial cells	Cytochrome *c*, caspases 3/7, 8 and 9	Schwarzer *et al*., 2012

Knowing that *P. aeruginosa* is able to induce apoptosis through its QS systems molecule, studies focusing on apoptosis induction should be considered with the context of QS signalling. Particularly, QS considerations should be taken into account when comparing studies using different multiplicity of infection or bacteria preparation protocols, processes that influence QS molecules concentration and/or bacteria population numbers.

## Conflict of interest

None declared.
